# Attention Deficit/Hyperactivity Disorder Symptoms Impair Adaptive and Social Function in Children With Autism Spectrum Disorder

**DOI:** 10.3389/fpsyt.2021.654485

**Published:** 2021-12-22

**Authors:** Yu Liu, Luxi Wang, Shu Xie, Shixu Pan, Jingyi Zhao, Mingyang Zou, Caihong Sun

**Affiliations:** ^1^Department of Children's and Adolescent Health, Public Health College, Harbin Medical University, Harbin, China; ^2^Chongqing Center for Disease Control and Prevention, Chongqing, China

**Keywords:** autism spectrum disorder, attention deficit/hyperactivity disorder, adaptive behavior, social function, comorbidity

## Abstract

**Background:** Autism spectrum disorder (ASD) often co-exists with attention deficit/hyperactivity disorder (ADHD), which may aggravate functional impairment. However, it is unclear how comorbid ADHD symptoms influence the adaptive behavior and social interaction deficits of children with ASD.

**Methods:** The study enrolled 340 children (ranging from 2 to 14 years) with ASD, with comorbid ASD and ADHD, or with typical development (TD). A psychological evaluation involving adaptive behavior and social function was conducted using the Vineland Adaptive Behavior Scale, Second Edition (VABS-II) and the Social Responsiveness Scale (SRS).

**Results:** There was a high prevalence of ADHD symptoms (46.6%) in children with ASD, and children with ASD + ADHD presented the worse profile of ASD symptoms. The ASD + ADHD group had higher scores on VABS and lower scores on SRS in comparison with the ASD alone group and TD group. The regression analysis revealed that ASD symptoms and ADHD symptoms were significantly associated with greater impairments in adaptive behavior and social function. The ADHD symptoms were responsible for an additional 0.8% of the variance in adaptive behavior, and 9.5% of the variance in social function.

**Conclusions:** More severe ASD symptoms and greater impairment in adaptive function and social ability were found in children with ASD and comorbid ADHD, highlighting the need to identify ADHD comorbidities early on in children with ASD and to reduce their negative impact on functioning.

## Introduction

Autism spectrum disorders (ASD) are behaviorally defined as neurodevelopmental disabilities, which are characterized by deficits in reciprocal social interaction and communication as well as by restricted, repetitive patterns of behavior or interests (1, 2). Recently, the Autism and Developmental Disabilities Monitoring (ADDM) Network reported that 1 in 54 children aged 8 years in the USA is diagnosed with ASD (3), which is an ~10% increase from the previous prevalence estimate (4). ASD often co-exists with other neuropsychiatric diseases (5, 6), such as attention deficit/hyperactivity disorder (ADHD), epilepsy, anxiety disorders, affective disorders, gastrointestinal symptoms, and sleep problems. These comorbidities have complex clinical manifestations and high lifetime prevalence, which could aggravate functional impairment and increase the burden of disease.

ADHD is one of the most commonly diagnosed childhood neurodevelopmental disorders, and its symptoms are mainly represented as inattention, hyperactivity, and impulsivity. According to a survey in 2016, an estimated 6.1 million US children 2–17 years of age (9.4%) had ever received an ADHD diagnosis (7). ADHD is often first identified in school-aged children when it negatively affects school achievement and family, peer and social interactions (8). According to the *Diagnostic and Statistical Manual for Mental Disorders, Fifth Edition* (DSM-5) in 2013, clinicians are able to make a diagnosis of ADHD in the context of ASD. This has led to increasing interest in the overlap of the clinical presentations of ASD and ADHD symptoms. Epidemiological studies have identified that the comorbidity rate of ADHD in ASD ranges from 14 to 70% in different countries (7, 9–11). To date, lines of work suggest there is a greater degree of cognitive impairment, poorer outcomes, and higher rates of internalizing and externalizing problems in children with comorbid ASD and ADHD than in those with ASD alone (12–14).

Adaptive behavior and social function are the most important measures in evaluating functioning in ASD. Adaptive behavior refers to the ability to perform the daily activities required for individual and social sufficiency (15), and is measured by the extent to which an individual can independently demonstrate developmentally appropriate practical skills, conceptual skills, and social skills (16). A cross-sectional study demonstrated that children and adolescents with ASD showed lower levels of overall adaptive functioning, as scored by the Vineland Adaptive Behavior Scale (VABS) (17). Individuals with ADHD also exhibit reduced adaptive functioning relative to their intelligence quotients (IQ)–matched typically developing (TD) group (18). It has been pointed out that patients with ASD show greater impairment of adaptive function than those with ADHD (19). Social-communication difficulties, a hallmark of ASD, are also observed in patients with ADHD, although impairment of social function is not part of the diagnostic criteria of ADHD (1). Moreover, Craig et al. (19) demonstrated the presence of social functioning impairment in patients with ADHD and characterized a symptomatic profile qualitatively similar to that of ASD, but of lower intensity. A Spanish study also indicated that the ASD group had the worst socialization skills. However, Ng et al. failed to find statistically significant differences in parent-reported social skills between an ASD group and an ADHD group (20). In general, there is a paucity of studies on the differences of adaptive behavior and social function between children with ASD and children with ASD and comorbid ADHD. The prior work did not clearly explain how comorbid ADHD symptoms influences the adaptive behavior and social interaction deficits of children with ASD.

Therefore, this study aims at clarifying the effect of ADHD symptoms in the context of ASD on adaptive behaviors and social function, and to analyze the independent effects of the ADHD symptoms on functional impairment in ASD children. We hypothesized that the children and adolescents with comorbid ASD and ADHD may exhibit more severe autism symptomatology, lower adaptive skills, and poor social function. Here, the present study recruited a case-control sample of children and adolescents (2–14 years old) with confirmed ASD, ASD with ADHD, and TD. This study would provide a more comprehensive understanding of the functional impairments in ASD with comorbid ADHD, and offer basic evidence for the development of personalized treatment training programs for individuals with ASD comorbidities.

## Method

### Participants

This cross-sectional study included 340 children, ranging from 2 to 14 years, who were consecutively recruited from the general public and the Child Developmental and Behavior Center of Harbin Medical University, Harbin, China. Diagnosis of ASD and/or ADHD was based on two independent specialist clinicians from our research team using the diagnostic criteria specified in DSM-5. The current gold standard for ASD diagnosis is based on clinical observation, substantiated by standardized testing of the patient with the Autism Diagnostic Interview-Revised (ADI-R) and the Autism Diagnostic Observation Schedule (ADOS). Diagnoses of ADHD were also confirmed by using the Vanderbilt ADHD Parent Rating Scale (VADPRS). Exclusion criteria included (1) neurodevelopmental disorders of known etiology (e.g., Fragile X Syndrome, tuberous sclerosis, or known chromosomal abnormalities or metabolic disorders), (2) significant sensory or motor impairment, and (3) serious chronic diseases. The neurodevelopmental histories and mental states of the typical development (TD) group were also evaluated and confirmed by neuropediatric specialists. The sample description and scores on primary measures were showed in Table 1.

**Table 1:** Demographic and clinical characteristics of the study groups.

	**ASD group**	***n***	**ASD + ADHD group**	***n***	**TD group**	***n***	***P***
Age (years)	5.77 ± 2.73	134	5.86 ± 2.86	117	5.97 ±2.23	89	0.862^a^
Gender (male: female)	110:24	134	101:16	117	82:7	89	0.104^**c**^
IQ	57 (40–72)	129	49 (38–60)	116	98 (81–114)	89	**<0.001^b^**
ADI-R	45.67 ± 10.47	123	49.18 ± 6.93	113	–		**0.003^d^**
ADOS	6.56± 1.42	131	7.13 ± 1.49	115	–		**0.002^d^**
CARS	32.79 ± 4.50	121	33.02 ± 4.10	107	–		0.694^**d**^
ABC	65.14 ± 34.52	121	82.05 ± 31.540	107	–		**<0.001^d^**
VADPRS	5 (3–7)	134	12 (10–14)	117	1 (0–4)	89	**<0.001^b^**
**VABS-II domains**							
Communication	77 (63–104)	134	69 (57–87)	113	113 (91–129)	89	**<0.001^b^**
Daily living skills	69 (60–89.75)	134	64 (58–75)	113	105 (84–123)	89	**<0.001^b^**
Socialization	60.5 (57–76)	134	58 (56–64)	113	110 (85–140)	89	**<0.001^b^**
Motor skills	87.5 (74–101)	134	82 (71–99)	113	100 (94–118)	89	**<0.001^b^**
Composite scale	68.5 (59–88.75)	134	62 (57–69)	113	106 (91–145)	89	**<0.001^b^**
**SRS**							
Social awareness	7.08 ± 5.22	130	8.03 ± 5.83	116	4.14 ± 4.53	87	**<0.001^a^**
Social cognition	16.01 ± 4.97	130	20.06 ± 4.25	116	11.08 ± 4.19	87	**<0.001^a^**
Reciprocal social interaction	26.97 ± 8.83	130	36.57 ± 6.84	116	14.09 ± 7.65	87	**<0.001^a^**
Social motivation	13.65 ± 4.71	130	16.34 ± 4.69	116	9.47 ± 4.43	87	**<0.001^a^**
Autistic mannerisms	11.13 ± 5.77	130	16.39 ± 5.40	116	6.57 ± 4.73	87	**<0.001^a^**
Total scale	74.59 ± 23.22	130	97.66 ± 18.30	116	45.36 ± 20.29	87	**<0.001^a^**

*Quantitative variables presented as mean ± standard deviation or median values (first and third quartiles)*.

a *one-way analysis of variance (ANOVA) followed by post-hoc least-significant difference (LSD) contrasts for multiple comparisons*;

b *Kruskal-Wallis H test and differences between two groups were assessed by Mann-Whitney U-test for post-hoc analysis*;

c *Pearson's chi-squared test*;

d *Independent samples t-tests. TD, typical development, IQ, intelligence quotient; ABC, Autism Behavior Checklist; CARS, Childhood Autism Rating Scale; ADOS, Autism Diagnostic Observation Schedule-Calibrated Severity Score; ADI-R, Autism Diagnostic Interview-Revised; VADPRS, the Vanderbilt ADHD Parent Rating Scale; VABS-II, Vineland Adaptive Behavior Scale; second edition; SRS, Social Responsiveness Scale. Bold values indicate P < 0.05*.

All participants recruited for the purpose of the research received oral feedback and a written summary of the results of evaluation without any payment. Caregivers provided written informed consent and children provided assent if possible. The procedures related to this study were approved by the Institutional Review Board of Harbin Medical University.

### Measures

In this study, all psychologists or trainees involved in administering the measurement protocols had established research reliability. Participants were evaluated using the ADI-R, ADOS, VAPRS, Vineland Adaptive Behavior Scale; Second Edition (VABS-II), Social Responsiveness Scale (SRS), Childhood Autism Rating Scale (CARS), Autism Behavior Checklist (ABC) and a cognitive assessment. Due to applicability, cooperativity and compliance from patients and their guardians, some cases did not complete all above examinations. Multiple assessments were available for many cases in this corpus. However, only the most recent assessment was retained for each case.

### ASD/ADHD-Specific Symptoms Assessment

The ADI-R (21) is a semi-structured interview administered to parents, designed to make a diagnosis of autism according collects information concerning both the child's current behavior and past behavior (i.e., most abnormal 4–5 scores or ever scores). Diagnosis of ASD was made on meeting the cutoff points of the ADI-R algorithm scores in the three domains: social interaction, communication, and restricted repetitive behavior. Autism severity was assessed based on the level of the scores; higher scores in each ADI-R subdomain reflected more severe autism symptoms.

The ADOS (22) is an interactive semi-structured assessment instrument designed to obtain information about autism features (in communication, reciprocal social interaction and repetitive or restricted behavior domains) in children. Assessment consists of a range of activities and social presses providing a standardized context in which to observe specific behaviors. The ADOS diagnostic algorithm yields calibrated severity scores (CSS) for the algorithm total (ADOS-CSS) which quantifies ASD symptom severity and higher ADOS-CSS scores reflect more severe autism symptoms. The CSS is a standardized score of the relative severity of autism-specific behaviors, is less influenced by participants demographics than by raw scores (23). Therefore, ADOS score in this study refer to ADOS-CSS.

The Childhood Autism Rating Scale (CARS) (24) was use to provide an estimate of autism severity. AS a standardized scale that evaluates the intensity of autism symptoms across 15 domains, each scored from 1 to 4. The total score is the sum of each of the 15 sub-scores (range 15–60), and a higher score indicating higher severity.

The ABC (25) was filled out by their parents and serves to screen for a number of behaviors. There are 57 sub items in the ABC, each one being scored; generally, the higher scores represent more serious behavioral problems.

The VADPRS was scored using the National Institute for Children s Health Quality s criteria, and consists of 45 statements on children s behaviors, 9 of which correspond with behaviors included in the Diagnostic and Statistical Manual of Mental Disorders for the predominately inattentive subtype of ADHD and 9 to the hyperactive/impulsive subtype. Higher scores reflect more severe of problem behavior of children with ADHD. This questionnaire had good reliability and validity (26, 27), and is recommended by the American Academy Pediatrics.

### Adaptive Behavior and Social Function Assessment

The VABS-II, is a measure of adaptive behavior based on parent interview with validity and reliability from birth to adulthood, and commonly incorporated in assessments for ASD. The VABS yields domain scores in the areas of communication, daily living skills, socialization, and motor skills, as well as an overall adaptive behavior composite. Lower scores indicated greater impairment of adaptive behavior. The VABS-II has demonstrated strong reliability and validity (15). In the current study, the Cronbach's alpha for the scale was 0.952.

The SRS (28) is a 65-item parent-completed questionnaire about autistic traits over the previous 6 months in 4 to 18-year-olds, which is categorized into five dimensions: social awareness, social cognition, reciprocal social interaction, social motivation and autistic mannerisms. Each item is scored from not true to almost always true. The usage of raw scores (higher scores indicated more serious impairment of social functions) is recommended for research. The Chinese Mandarin version of the scale had adequate reliability and validity (29). In the current study, the Cronbach's alpha for the scale was 0.916.

### Cognitive Assessment

The Wechsler Preschool and Primary Scales of Intelligence (30) or Wechsler Intelligence Scale for Children (31), are used to evaluate intellectual functioning and determine the IQ. Standardized estimates of verbal IQ, performance IQ, and full-scale IQ were derived using IQ norms with *M* = 100 and SD = 15. In this study, we use the full scale IQ as an indicator of IQ.

### Statistical Analyses

Comparisons between groups were examined, as appropriate, by means of independent samples *t*-tests or one-way analyses of variance (ANOVA), followed by *post-hoc* Welch two-sample *t*-test and Least-significant (LSD). To non-normal distribution of data, multiple comparisons among three groups were conducted by Kruskal-Wallis H test and differences between two groups were assessed by Mann-Whitney *U*-test for *post-hoc* analysis. Pearson's chi-squared test was used to compare gender composition between groups. Linear regression model with enter method (VABS and SRS) as outcome variable was conducted to test the associations of potential explanatory variables. The demographics (gender, age, and IQ) as covariates were entered into the first block, followed by ASD diagnosis in the second block and ADHD diagnosis in the third block. The model R^2^ is reported as interpretation of the meaning of such individual coefficients is limited by multicollinearity. Significance level was set at *p* < 0.05 (two-tailed). SPSS Version 21.0 (SPSS Inc., Chicago, IL, USA) was used for all statistical analyses.

## Results

Overall, the 340 children in our study included 293 boys and 47 girls with a mean age of 5.85 ± 2.65 years: 134 children with ASD (ASD group), 117 children with ASD and ADHD (ASD + ADHD group) and 89 with typical development (TD group). The ADHD comorbidity rate was 46.6% in the children with ASD. As shown in Table 1, the mean age and gender ratio did not significantly differ among the three groups (*F* = 0.149; *p* = 0.862 and χ^2^ = 4.533; *p* = 0.104). However, IQ was significantly different among the groups (χ^2^ = 143.574; *p* < 0.001), and the *post-hoc* analyses showed that IQ was significantly lower in the ASD + ADHD group, compared with the TD group (*p* < 0.001) and the ASD group (*p* < 0.013). Meanwhile, there was significantly lower IQ in the ASD group, compared with the TD group (*p* < 0.001).

**Figure 1:**
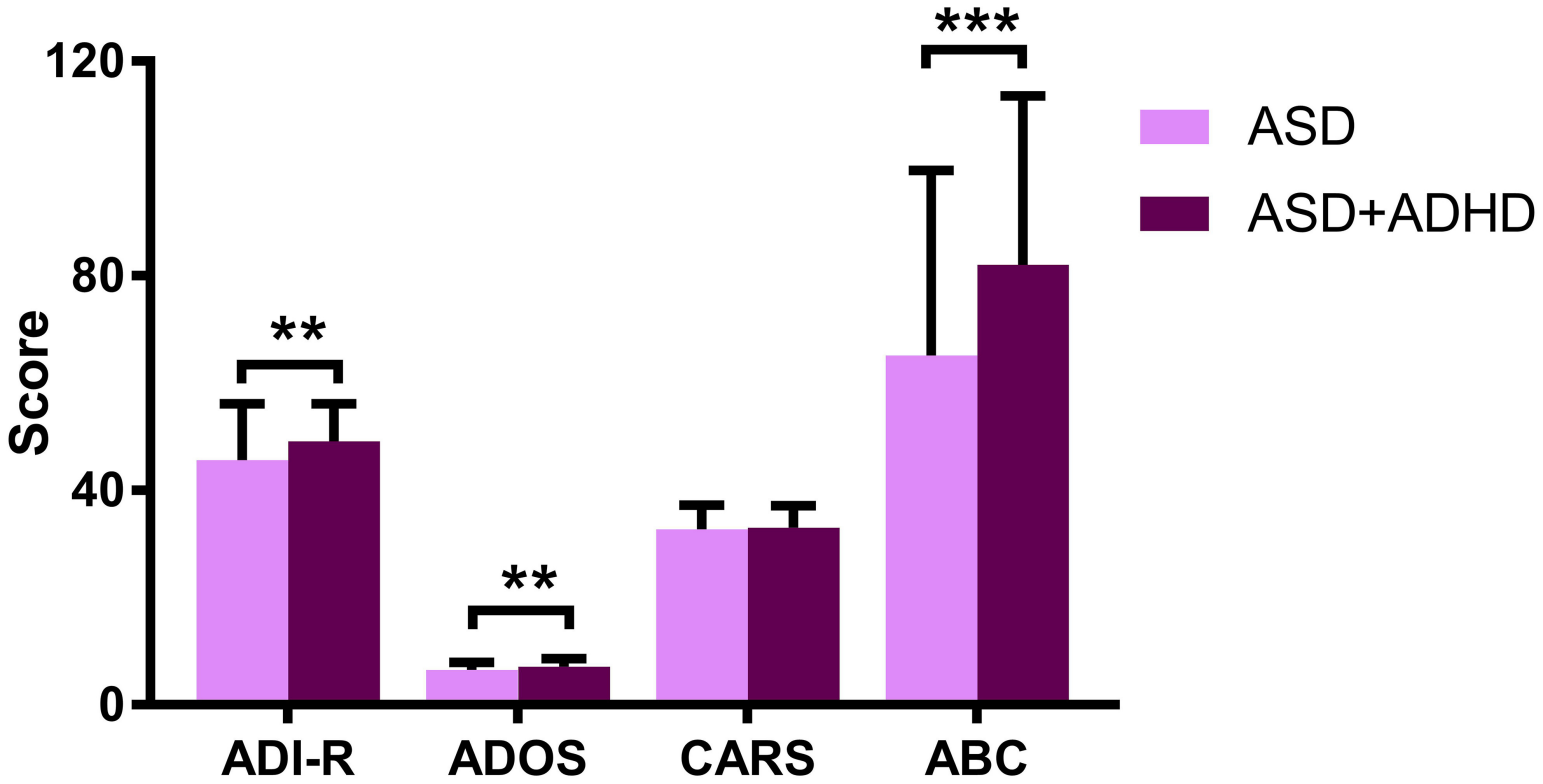
Graph displaying the profile of the scores of each diagnostic group for the ADI-R, ADOS, CARS, and ABC. **P < 0.01; ***P < 0.001.

The ASD + ADHD group had higher scores than the ASD group in the ADI-R (*t* = −3.052; *p* = 0.003), ADOS (*t* = −3.086; *p* = 0.002), and ABC (*t* = −3.842; *p* < 0.001). However, the scores for the CARS were not significantly different between the two groups (*t* = −0.393; *p* = 0.694) (Table 1; Figure 1).

**Figure 2:**
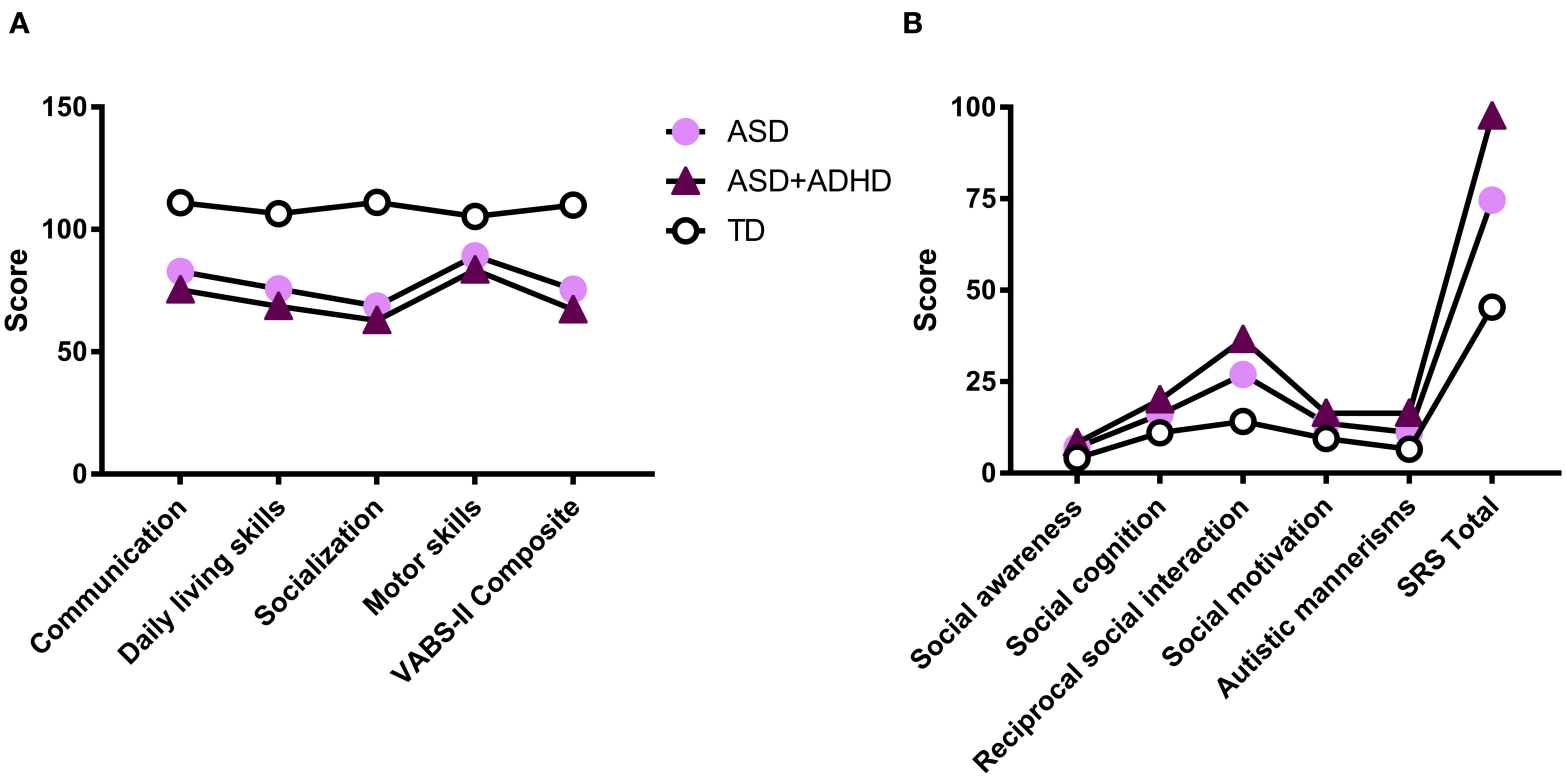
Graphs displaying the profile of scores of each diagnostic group on the VABS-II **(A)** and SRS **(B)**.

In the VABS-II, we found significant differences among the three groups across all domains and combined scores: Communication (χ^2^ = 84.932; *p* < 0.001), Daily living skills (χ^2^ = 106.643; *p* < 0.001), Socialization (χ^2^ = 143.458; *p* < 0.001), Motor skills (χ^2^ = 50.009; *p* < 0.001), and the Composite scale (χ^2^ = 114.885; *p* < 0.001) (Table 1). The *post-hoc* analyses showed that, in all subscales, the scores of the ASD + ADHD group were significantly lower than those of the ASD group, and the scores of the ASD group were significantly lower than those of the TD group (Supplementary Table 1; Figure 2A).

In terms of social function, the SRS scores differed significantly among the groups: social awareness (*F* = 15.872; *p* < 0.001), social cognition (*F* = 97.641; *p* < 0.001), reciprocal social interaction (*F* = 202.399; *p* < 0.001), social motivation (*F* = 54.942; *p* < 0.001), autistic mannerisms (*F* = 84.074; *p* < 0.001), and SRS total scale (*F* = 156.382; *p* < 0.001) (Table 1). Subsequently, the *post-hoc* analyses showed that the scores of the ASD + ADHD group were significantly higher than those of the ASD group, and that those of the ASD + ADHD group and ASD group were significantly higher than those of the TD group in social cognition, reciprocal social interaction, social motivation, and autistic mannerisms domains, and for the SRS total. However, there were no statistically significant differences between the ASD group and the ASD + ADHD group in the social awareness domain (Supplementary Table 1; Figure 2B).

Supplementary Table 2 shows the bivariate correlations between dependent variable and the result variable. To identify the influence of ASD and ADHD symptoms on the VABS-II composite scale and SRS total scale, multiple linear regression analyses using enter methods were conducted (Table 2). The VABS-II and SRS scores were affected by demographic characteristics, with demographic characteristics explaining 40.3 and 34.1% of the variance, respectively.

**Table 2:** Linear regression models.

	**VABS-II (*****n*** **= 336)**				**SRS (*****n*** **= 323)**			
	**B**	**SE B**	**β**	***P***	**B**	**SE B**	**β**	***p***
**Model 1**								
Constant	**84.316**	**7.194**		**<0.001**	**62.268**	**7.166**		**<0.001**
Gender	3.965	3.311	0.048	0.232	−2.173	3.253	−0.026	0.504
Age	**−1.343**	**0.457**	**−0.124**	**0.004**	**0.936**	**0.444**	**0.085**	**0.036**
IQ	**0.342**	**0.057**	**0.343**	**<0.001**	**−0.229**	**0.057**	**−0.222**	**0.001**
***R*****^2^**	**0.403**				**0.341**			
**Model 2**								
**ASD diagnosis**	**−21.608**	**3.621**	**−0.342**	**<0.001**	**20.830**	**3.640**	**0.316**	**<0.001**
***ΔR*****^2^**	**0.073**				**0.096**			
**Model 3**								
**ADHD diagnosis**	**−6.095**	**2.651**	**−0.103**	**0.022**	**21.160**	**2.626**	**0.347**	**<0.001**
***ΔR*** **^2^**	**0.008**				**0.095**			

*Outcome variables: VABS-II and SRS. Bold = P < 0.05. Value: ASD, 1; not ASD, 0; ADHD, 1; not ADHD, 0. SE, standard error*.

After controlling for demographic confounders (gender, age, and IQ), for VABS-II, when the ASD diagnosis variable was added (model 2), 7.3% of the variance was explained by ASD diagnosis; when the ADHD diagnosis variable was further added (model 3), the R^2^ value increased by 0.8% (*F* = 60.855; *p* < 0.001), which was statistically significant. The ASD diagnosis (β = −0.342; *p* < 0.001) and ADHD diagnosis (β = −0.103; *p* = 0.022) were negatively correlated with the VABS-II score. For the SRS, when the ASD diagnosis variable was added alone (model 2), it explained 9.6% of the variance in the SRS scores; when the ADHD diagnosis variable was further added (model 3), the R^2^ value increased by 9.5% (*F* = 72.941; *p* < 0.001), which was statistically significant. The ASD diagnosis (β = 0.316; *p* < 0.001) and ADHD diagnosis (β = 0.347; *p* < 0.001) were significantly positively correlated with the SRS score.

## Discussion

In this population-based case-control study, we found a high prevalence of ADHD symptoms (46.6%) in children with ASD. Children with ASD + ADHD exhibited more severe ASD traits, and a greater degree of weakness in adaptive profile and social function. Moreover, ADHD symptoms could aggravate the impairment of adaptive behavior and social function in individuals with ASD.

Previous research (32, 33) has found that the subgroup of individuals with ASD without other psychiatric comorbidities showed less severe autistic symptoms (scored by ADIR) in comparison with the subgroup with ASD with ADHD, while they failed to find significant differences in the ADOS. Notably, there may be bias in the evaluation of autism severity from parental reports (i.e., ADI-R and ABC) and professionals' observations (i.e., ADOS and CARS). Interestingly, this study found significant differences in both parental reports (ADI-R and ABC) and professionals' observations (ADOS). The explanation for the discrepancy is that, in the study of Sprenger et al. (32), they did not apply the CSS, a standardized indicator of autism severity from the ADOS. Avni et al. (33) compared the ADOS subdomains: social affect-CSS and restricted repetitive behavior-CSS but not the ADOS total CSS of the present study. In addition, there may be a possible measurement bias due to the different psychologists or trainees involved in our current study and others. Overall, these studies were consistent with our findings that individuals with ASD and ADHD have a tendency toward more severe ASD-like features when compared with those with only ASD.

In extrapolating from the current data, children with ASD + ADHD had worse performance in adaptive behaviors than children with only ASD and children with TD, which were noted in the communication, daily living skills, socialization and motor skills subdomains, and in the VABS composite. The findings of the present study were in line with previous reports and provide supportive evidence that ADHD symptoms have an impact on adaptive behaviors (33, 34). Avni et al. (33) reported that children in their ASD + ADHD group had greater impairments in the socialization subdomain of adaptive behaviors. The Autism Treatment Network program of Autism Speaks also documented that the ASD + ADHD group was significantly more impaired in adaptive functioning in all the VABS subdomains as compared with the ASD alone group (34). Indeed, compared with children with only one of these diseases, individuals with ASD and ADHD encounter more difficulties in daily life, bear greater impaired adaptive function and experience poorer quality of life (34, 35). The raw scores of VABS-II could be transferred into standard scores according to assessment criteria. However, the subscale and total SRS scores are based on raw scores and the scores of five subscale are added as total scores. Obviously, the scores of the reciprocal social interaction subscale in in the ASD + ADHD are extremely prominent, which can drive the total score to a certain extent. It has been reported that there is more impairment in social interaction in children with ASD comorbid with ADHD than in children with ASD alone (12). In the clinical guidelines developed by the National Institute of Health and Care Excellence in London for children with ASD, social communication interventions are taken as specific core features interventions (36). After controlling for confounders (i.e., age, socioeconomic status, gender ratio, and IQ), it was also found that children in the ASD + ADHD group received a higher score in the SRS, and showed more severe impairment of social communicative functions (37). The prior research has indicated that the SRS total scores of children with ADHD were intermediary between those of children with ASD and those of TD children (20). Additionally, the present study found that children with ASD + ADHD had higher SRS total scores than those with ASD and TD children, and that greater impairment was observed in social cognition, reciprocal social interaction, social motivation, and autistic mannerisms. At this point, we suggest that the coexistence of ADHD may constitute a distinctive phenotype of ASD, and these children may be at higher risk of adaptive problems and social impairment.

A large-scale population-based study demonstrated that ASD symptoms explained 18% of the variance in adaptive behavior, while the same variables were responsible for 14% of the variance in social function (33). This study also found that the ASD symptoms were responsible for 7.3% of the variance in adaptive behavior, vs. 9.6% accounted for when utilizing an identical model predicting social function. It is observed that ASD symptoms have a similar effect on adaptive behavior and social function in individuals with ASD. Another result of the present study was that ADHD symptoms explained 0.8% of the additional variation in adaptive behavior and 9.5% of the additional variation in social function. The ADHD symptoms were significantly associated with greater impairments in adaptive behaviors and social function. In particular, Avni et al. (33) revealed that the inattention associated with ADHD symptoms, not the hyperactivity/impulsivity, was attributed to lower adaptive behavior. This could account for the relatively low proportion of variance in adaptive behavior explained by ADHD symptoms in the current study, because the current study did not further divide the ADHD symptoms, and used the total severity of ADHD symptoms instead. The abovementioned findings also serve as a reminder of the importance of early identification of comorbidities and providing effective intervention to reduce their negative impact on adaptive behavior and social function.

One potential explanation for the findings is that symptoms of comorbidity will confuse the diagnosis of ASD, so that when ASD is diagnosed, the symptoms are more severe and the function is more impaired than in other children of the same age. For example, a study from the National Survey of Children's Health found that ~20% of 1,500 U.S. children aged 2–17 years with parent-reported ASD were initially given a diagnosis of ADHD, and were diagnosed with ASD three years later than children without ADHD concerns (7). Our IQ results were consistent with Rau et al. (38), compared with ASD alone, comorbid ADHD have more severe IQ impairment. These children and adolescents with poorer social skills and lower IQ may have weak awareness of social differences. On the other hand, comorbid ADHD symptoms may put the children and adolescents at greater levels of non-compliance, and aggression, which in turn affects the parental-reported adaptive functioning (39). Furthermore, it is worth noting that the symptoms of ADHD, including persistent inherent negligence and poor impulse control, would obviously bring about negative effects on the treatment efficacy of intervention training in ASD + ADHD children. Antshel et al. (40) recruited children with ASD and children with ASD and comorbid ADHD to participate in the same social skills training. They found that the social skills of children with ASD could be improved, while the treatment did very little to help children with ASD and comorbid ADHD. Therefore, it is necessary to identify the subgroup of children with ADHD at an early stage in the course of ASD, and to provide personalized treatment plans, especially in adaptive behavior and social interaction training. This will be conducive to the functional recovery of children with ASD accompanied by ADHD, and better promote their early return to mainstream society.

In addition, it is well-known that the SRS and VABS assessment outcomes are affected by the individual characteristics of children, such as age, gender and cognitive level (23). The European Autism Project cohort found that older age and lower level of cognitive ability were associated with more severely impaired adaptive function (41). Moreover, relative weaknesses in daily living skills were found for females compared with males diagnosed with ASD (42). Although a higher SRS score indicates more severe social deficits, research has indicated that when using the SRS as a quantitative phenotype measure, the influence of age and cognitive level on the scores must be considered (43). The research by Havdahl et al. (44) also pointed out that valid interpretation of SRS measurements requires steps to account for the influence of IQ. Based on this, in the current study, we demonstrated the effect of ASD symptoms and ADHD symptoms on adaptive behavior and social interaction more accurately by adjusting for these confounding factors.

There are some limitations to the current research. First, we did not enroll children with only ADHD in the analysis, and the study could have benefited from comparisons with the ADHD population. Second, there is a selection bias in our research. On the one hand, all participants involved in this study were from Heilongjiang Province, on the other hand, fewer older children are recruited, so it is difficult to extrapolate the results to the general population. Finally, whether the children with ASD and ADHD received medical treatment for ADHD was not taken into account. For a more detailed understanding of the changes of adaptive function and social function in ASD, a longitudinal study is needed to explore further the differentiated executive strategies required in the interventions designed for children with ASD and ADHD.

## Conclusion

In summary, our results indicate that, for individuals with ASD who are diagnosed with ADHD, the characteristics of the comorbidity made their ASD symptoms more severe, and exacerbated their deficits in adaptive function and social ability. In light of the these findings, it is important to identify ADHD comorbidities early on in children with ASD, and to explore effective medical and behavioral interventions to reduce the impact of ADHD symptoms, thereby hopefully leading to improved functioning.

## Data Availability Statement

The original contributions presented in the study are included in the article/Supplementary Material, further inquiries can be directed to the corresponding author/s.

## Ethics Statement

The studies involving human participants were reviewed and approved by Harbin Medical University. Written informed consent to participate in this study was provided by the participants' legal guardian/next of kin. Written informed consent was obtained from the individual(s), and minor(s)' legal guardian/next of kin, for the publication of any potentially identifiable images or data included in this article.

## Author Contributions

MZ and CS conceived and designed the research. YL, LW, SX, SP, and JZ performed clinical assessment. YL analyzed the data and wrote the paper. CS and MZ contributed reagents, materials, and analysis tools. All authors contributed to the article and approved the submitted version.

## Conflict of Interest

The authors declare that the research was conducted in the absence of any commercial or financial relationships that could be construed as a potential conflict of interest.

## Publisher's Note

All claims expressed in this article are solely those of the authors and do not necessarily represent those of their affiliated organizations, or those of the publisher, the editors and the reviewers. Any product that may be evaluated in this article, or claim that may be made by its manufacturer, is not guaranteed or endorsed by the publisher.
