# Supplementary material for: Attention Deficit/Hyperactivity Disorder Symptoms Impair Adaptive and Social Function in Children With Autism Spectrum Disorder
Source: Front Psychiatry. 2021 Dec 22;12:654485. doi: 10.3389/fpsyt.2021.654485 (PMC8727694; doi:10.3389/fpsyt.2021.654485)
Supplement: Supplementary file 1 [file Data_Sheet_1.docx]

**Table S1** Post-hoc analyses for IQ, VADPRS, VABS-II, and SRS

| Domains | ASD vs. ASD+ADHD |  | ASD vs.  NT |  | ASD+ADHD vs. NT |
| --- | --- | --- | --- | --- | --- |
|  | *p* |  | *p* |  | *p* |
| IQ | 0.102 |  | < 0.001 |  | < 0.001 |
| VADRS | < 0.001 |  | < 0.001 |  | < 0.001 |
| VABS-II domains |  |  |  |  |  |
| Communication | 0.046 |  | < 0.001 |  | < 0.001 |
| Daily living skills | 0.053 |  | < 0.001 |  | < 0.001 |
| Socialization | 0.036 |  | < 0.001 |  | < 0.001 |
| Motor skills | 0.044 |  | < 0.001 |  | < 0.001 |
| Composite scale | 0.019 |  | < 0.001 |  | < 0.001 |
| SRS |  |  |  |  |  |
| Social awareness | 0.072 |  | < 0.001 |  | < 0.001 |
| Social cognition | < 0.001 |  | < 0.001 |  | < 0.001 |
| Reciprocal social interaction | < 0.001 |  | < 0.001 |  | < 0.001 |
| Social motivation | < 0.001 |  | < 0.001 |  | < 0.001 |
| Autistic mannerisms | < 0.001 |  | < 0.001 |  | < 0.001 |
| Total scale | < 0.001 |  | < 0.001 |  | < 0.001 |

*IQ, intelligence quotient; ABC, Autism Behavior Checklist; CARS, Childhood Autism Rating Scale; ADOS, Autism Diagnostic Observation Schedule–Calibrated Severity Score; ADI-R, Autism Diagnostic Interview–Revised; VADPRS, the Vanderbilt ADHD Parent Rating Scale; VABS-II, Vineland Adaptive Behavior Scale; second edition; SRS, Social Responsiveness Scale.*

**Table S2** The correlational coefficients between study variables.

|  | 1 | 2 | 3 | 4 | 5 | 6 | 7 | 8 |
| --- | --- | --- | --- | --- | --- | --- | --- | --- |
| 1. **Gender** | - |  |  | - |  |  |  |  |
| 2. **Age** | -0.018 | - |  |  |  |  |  |  |
| 3. **IQ** | -0.037 | -0.211***** | - |  |  |  |  |  |
| 4. **VADPRS** | 0.020 | -0.040 | 0.424***** | - |  |  |  |  |
| 5. **ADI-R** | 0.013 | 0.476***** | -0.291***** | 0.232***** | - |  |  |  |
| 6. **ADOS** | 0.043 | 0.004 | 0.442***** | 0.228***** | 0.217***** | - |  |  |
| 7. **VABS-II** | 0.004 | -0.206***** | 0.726***** | -0.486***** | -0.306***** | -0.460***** | - |  |
| 8. **SRS** | 0.020 | 0.090 | -0.579***** | 0.681***** | 0.371***** | 0.300***** | -0.598***** | - |

*IQ, intelligence quotient; ADOS, Autism Diagnostic Observation Schedule–Calibrated Severity Score; ADI-R, Autism Diagnostic Interview–Revised; VADPRS, the Vanderbilt ADHD Parent Rating Scale; VABS-II, Vineland Adaptive Behavior Scale; second edition; SRS, Social Responsiveness Scale.*

****p* < 0.05**
